# Systematic analysis of *Histidine photosphoto *transfer gene family in cotton and functional characterization in response to salt and around tolerance

**DOI:** 10.1186/s12870-022-03947-5

**Published:** 2022-11-28

**Authors:** Lanjie Zhao, Liangqing Sun, Lixue Guo, Xuke Lu, Waqar Afzal Malik, Xiugui Chen, Delong Wang, Junjuan Wang, Shuai Wang, Chao Chen, Taili Nie, Wuwei Ye

**Affiliations:** 1 Institute of Cotton Research of Chinese Academy of Agricultural Sciences / Zhengzhou Research Base, State Key Laboratory of Cotton Biology, School of Agricultural Sciences, Zhengzhou University, Anyang, Henan, 455000 China; 2 Cotton Research Institute of Jiangxi Province, Jiujiang, Jiangxi, 332105 China

**Keywords:** *Histidine phosphotransfer*, Cotton, Salt stress, Drought stress, Two-component system

## Abstract

**Background:**

Phosphorylation regulated by the two-component system (TCS) is a very important approach signal transduction in most of living organisms. Histidine phosphotransfer (HP) is one of the important members of the TCS system. Members of the *HP* gene family have implications in plant stresses tolerance and have been deeply studied in several crops. However, upland cotton is still lacking with complete systematic examination of the *HP* gene family.

**Results:**

A total of 103 *HP* gene family members were identified. Multiple sequence alignment and phylogeny of HPs distributed them into 7 clades that contain the highly conserved amino acid residue “XHQXKGSSXS”, similar to the *Arabidopsis* HP protein. Gene duplication relationship showed the expansion of *HP* gene family being subjected with whole-genome duplication (WGD) in cotton. Varying expression profiles of HPs illustrates their multiple roles under altering environments particularly the abiotic stresses. Analysis is of transcriptome data signifies the important roles played by *HP *genes against abiotic stresses. Moreover, protein regulatory network analysis and VIGS mediated functional approaches of two *HP* genes (*GhHP23* and *GhHP27*) supports their predictor roles in salt and drought stress tolerance.

**Conclusions:**

This study provides new bases for systematic examination of *HP* genes in upland cotton, which formulated the genetic makeup for their future survey and examination of their potential use in cotton production.

**Supplementary Information:**

The online version contains supplementary material available at 10.1186/s12870-022-03947-5.

## Background

Adverse environmental conditions like salinity and drought stress, affect the plant growth development (PGD) and quality of seed very badly. For plant adaptability to these altering environments, they acquired a sensitive protection system in the process of evolution so that they can rapidly sense, respond to and adapt to these pressures appropriately [1–3]. Phosphorylation regulated by the two-component system (TCS) is a very important approach of signal transduction in most of living organisms. In plants, the TCS for signal transduction mainly consists of histidine kinases, histidine phosphotransfers and response regulators. HPs are the downstream target proteins of cytokinin receptor HKs, which receive and transfer the phosphate groups from the receptor HKs and transfer the phosphate groups to the downstream *Arabidopsis* response regulators (ARRs) to complete the downstream transduction of cytokinin signals. They have roles in important cellular mechanisms, for example, cytokinin responses, reaction to red light and ethylene, as well as osmosensing [4–7], suggesting their imperative implications in plant stress responses.

*Arabidopsis* contains 6 HP proteins. In AHP1-AHP5, there is a conserved phosphorylation site of the His residue similar to that in prokaryotes and yeast, and receives phosphate groups from the receptor AHK to complete the downstream transduction of the cytokinin signal [8]. However, AHP6 does not contain the conserved His residues, and it plays a negative regulatory role in the cytokinin signaling pathway by competing with AHP1-AHP5 to bind the ARR protein [9]. The molecular weights of AHPs are very small, and they are transported into and out of the nucleus actively, which is not related to their cytokinin level or phosphorylation status [10]. AHP2 and AHP5 are evenly distributed in the cytoplasm and nucleus, and cytokinin treatment does not change their subcellular localization [10].

Different *AHP* genes have different expression patterns. *AHP1* is mainly expressed in roots [8]; *AHP2*, *AHP3* and *AHP5* are widely expressed in various above and belowground tissues; and *AHP4* is mainly expressed in young floral organs [8, 11]. *AHP6* is significantly expressed in pericylindrical cells in the xylem and in aerial tissues [8, 9]. There is functional redundancy among the members of the *AHP* family [12, 13]. None of the *ahp* mutants showed significant cytokinin-related growth and development phenotypes. The *ahp1ahp2ahp3* and *ahp2ahp3ahp5* mutants have a phenotype that is not sensitive to exogenous cytokinins, and *ahp2ahp3ahp5* has a significant growth inhibition phenotype. The *ahp1ahp2-1ahp3ahp4ahp5* mutants have severe growth and development phenotypes, while the mutants with complete loss of *Ahp1ahp2-2ahp3ahp4ahp5* function also have female gamete abortion [14].

Studies have shown that modification of the AHP1 protein by S-nitrosylation can inhibit its own phosphorylation and that of its downstream component ARR1 protein, as well as negatively regulate cytokinin signaling [15]. This result indicates that the redox potential can directly affect the cytokinin regulation signals, thereby shows the involvement in regulation of PGD. The *ahp2ahp3ahp5* triple mutant has obvious defects in embryo sac development, and the central cell nuclear antipodal cells are absent in the ovule, homologus to the phenotype of *cki1*. This implies that AHP2, AHP3 and AHP5 may form a binary component with CKI1 and become the main factor promoting the female gametophytes development in *Arabidopsis* [16].

Cotton is not only an important cash crop worldwide but also a pioneer crop planted in saline-alkali soil. The roles of *AHPs* in salt and drought stress have been deeply studied, however, cotton is not well known for their functions, especially the mechanism of action during salt tolerance and drought stress. In current study, the characteristics of the *HP* gene family were examined and analyzed in cotton; and their expression in response to abiotic stresses and different tissues in *Gossypium hirsutum* L. were researched. The functions of *GhHP23* and *GhHP27* in response to drought and salt tolerance were investigated using VIGS technology. It was found the silencing of these two genes could improve the salt and drought tolerance of cotton. Our results offer useful acumens into the function of *HP* genes in cotton in the future, which may offer several *HP* candidate genes for resistance breeding in cotton.

## Results

### Identification of *HP* genes

BLASTp search of *HP* genes from *Oryza sativa* (*Os*) and *Arabidopsis* (*At*) used as query results into identification of a total of 30, 34, 21, 18, 10, 18, 9, 7 and 18 *HP* genes from *G. hirsutum* (*Gh*), *G. arboreum* (*Ga*), *G. barbadense* (*Gb*), *G. raimondii* (*Gr*), *Theobroma cacao* (*Tc*), *Populus trichocarpa* (*Pt*), *Vitis vinifera* (*Vv*), *Zea mays* (*Zm*) and *Glycine max* (*Gm*), respectively. The genes were named according to the position on the chromosome in ascending order: (*GhHP1-30*, *GbHP1-34*, *GaHP1-21*, *GrHP1-18*, *TcHP1-10*, *PtHP1-18*, *VvHP1-30*, *ZmHP1-34* and *GmHP1-18*). The detailed information is presented in Table S2.

In addition, we also analyzed the biophysical features of *HP* genes in cotton: like genomic, CDS and protein lengths, isoelectric point, molecular weight, and subcellular localization. Among the 103 identified cotton HP members, with fewer than 100 amino acids, *GaHP1* was found as the smallest HP protein among 103 total *HP* genes, and *GrHP1* was recognized as the largest HP protein. The molecular weight ranged from 7 to 25.41 kDa, whereas the pI ranged from 4.78 to 17.28. The predicted results of subcellular localization suggest that most of them are localized in the nucleus, but some HP proteins are localized in or only in the extracellular region or cytoplasm. It is speculated that HP proteins located in different cell compartments may perform different functions. The detailed information is presented in Supplementary Table S3.

### Evolutionary analysis of *HP* Gene family in multiple plant species

**Fig. 1:**
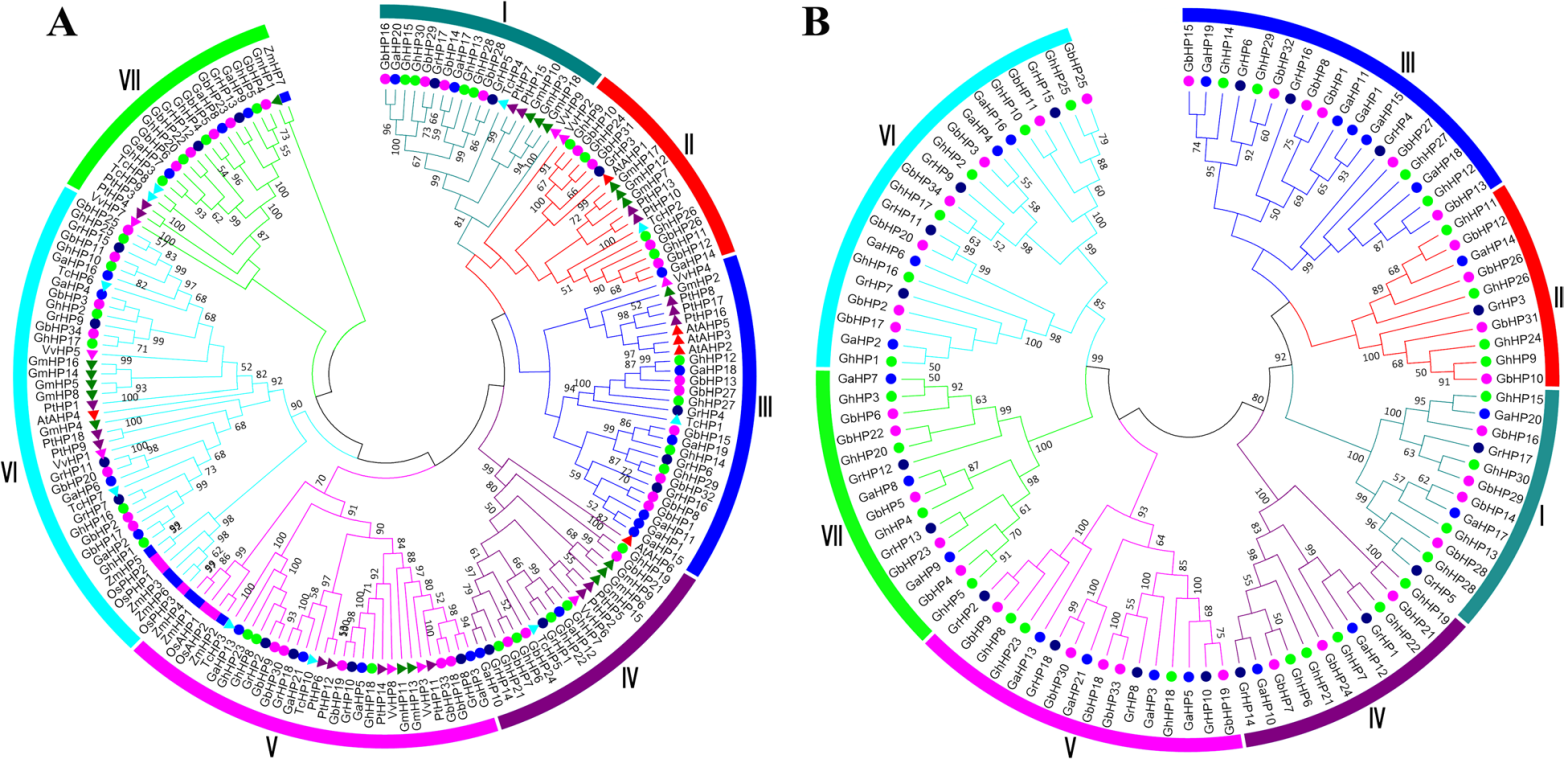
The phylogeny trees of *HP* family genes. **A** Phylogenetic relationship of the 171 identified *HP* genes from 5 plant species. *Arabidopsis thaliana* (*At*), *G. hirsutum* (*Gh*), *G. barbadense* (*Gb*), *G. arboreum* (*Ga*), *G. raimondii* (*Gr*), *Theobroma cacao* (*Tc*), *Populus trichocarpa* (*Pt*), *Vitis vinifera* (*Vv*), *Zea mays* (*Zm*) and *Glycine max* (*Gm*). **B** Phylogenetic relationship of 109 identified *HP* genes from four cotton species and *Arabidopsis*. The two neighbor joining (NJ) phylogeny trees constructed using MEGA 7.0 software. Bootstrap values above 50% from 1000 replicates are shown at each node

Multiple sequence alignment of the cotton HP proteins showed that there was a highly conserved amino acid residue sequence "XHQXKGSSXS"(Fig. S1, S2, S3 and S4). It is similar to the *Arabidopsis* HP protein. The conserved sequenses of the *HP* gene family members shows the great level of conservation between cotton and *Arabidopsis*. Phylogenetic tree analysis showed that 176 HP proteins of 11 plant species were divided into 7 subfamilies (Fig. 1).

The HP proteins in the V, VI and VII subfamilies of monocots clustered together separately, but there were no monocot HP proteins in I, II, III and IV subfamilies. This shows that the HP protein of dicotyledonous plants in I, II, III and IV subfamilies has expanded in the course of evolution. In addition, this was also illustrated in the phylogenetic tree: the protein number of allotetraploid cotton, *G. hirsutum* and *G. barbadense* is almost twice that of *G. arboreum* and *G. raimondii*, indicating that the HP protein has amplified in the process of evolution. Therefore, it is speculated that two tetraploid species (Gh, Gb) were originated from two diploid plant species (Ga, Gr) after their hybridization between the two diploid cotton species.

### Gene structure analysis and conserved motifs

**Fig. 2:**
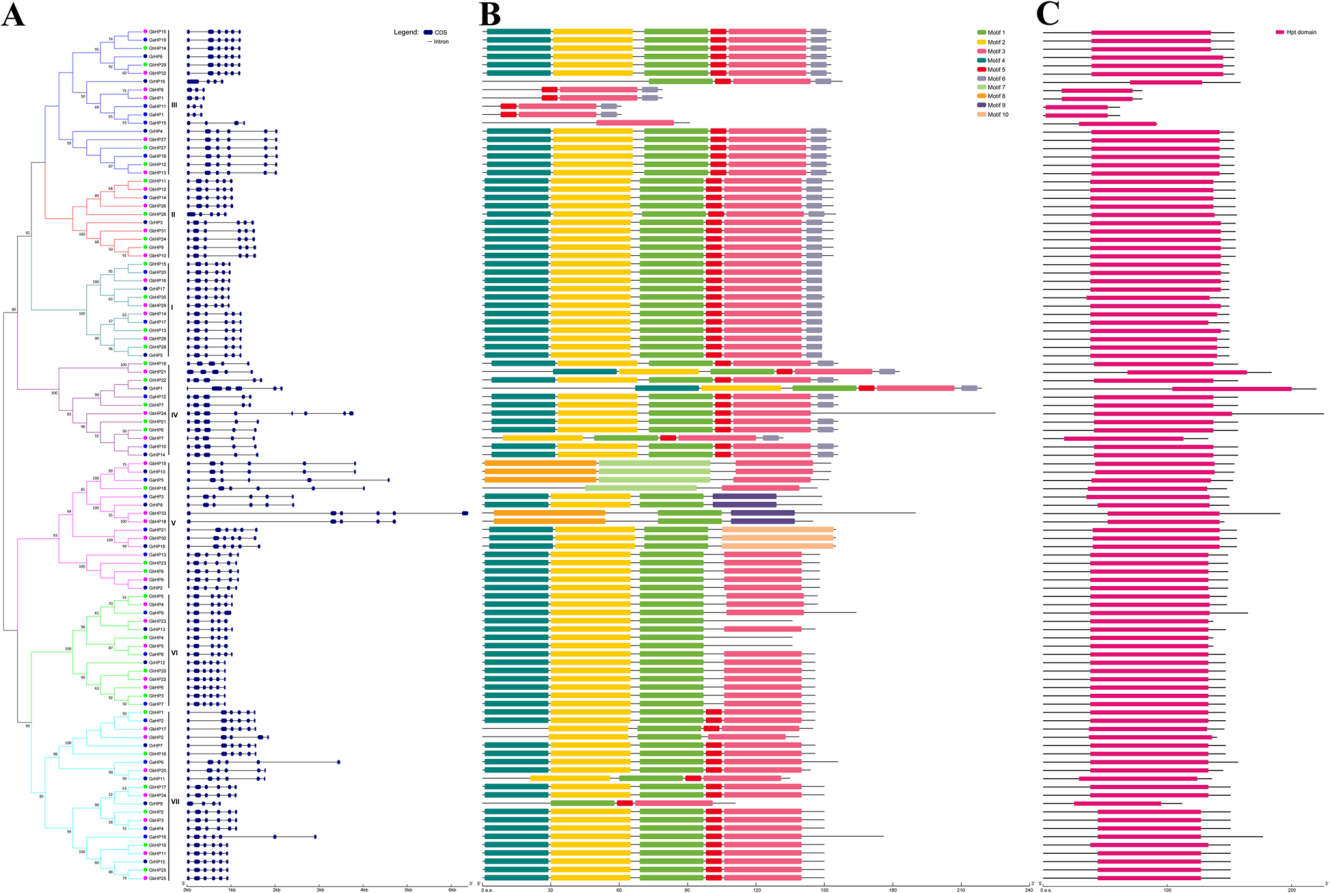
Comparison of the gene structure, conserved protein motifs and domains in *HP* genes on the *G. hirsutum*. **A** The NJ phylogenetic tree was constructed based on the full-length sequences of *G. hirsutum* HP proteins using MEGA 7.0 software, and Exon-intron structure of *G. hirsutum HP* genes. Details of subfamilies are shown in different colors. Blue boxes indicate exons; black lines indicate introns. **B** The motif composition of *G. hirsutum*. The motif numbers 1–10, are displayed in different colored boxes. **C** Schematic representation of the conserved domains in *G. hirsutum* HP proteins. The Hpt domain is highlighted by pink box. The length of DNA genomic or protein can be estimated using the scale at the bottom

In plants, the exons and introns of gene structure are related to their biological functions. The *HP* gene in cotton generally contains 6 exons and 5 introns in this study (Fig. 2A). The *GbHP24* gene in the IV subfamily contains the most exons (8) and introns (7), and the *GbHP8*, *GbHP1*, *GaHP11*, while *GaHP1* genes in the III subfamily contain the fewest exons (3) and introns (2). The motif analysis of the cotton HP proteins showed that 10 motifs were distributed on different cotton HP proteins (Fig. 2B). Most HP proteins contain 6 motifs, only *GaHP15* contains 1 motif (Motif 3), and *GhHP18* contains 2 motifs (Motif 3 and Motif 7). In addition, through the analysis of the cotton HP protein domains, we come to know that all HP proteins from cotton encompass a conserved HP domain, which is the basic feature of HP family proteins (Fig. 2C).

### Chromosomal distribution and collinear analysis

The chromosome position of the *HP* family genes in four cotton species was analyzed. The results showed that 103 *HP* genes were disseminated on different chromosomes (Fig. S5). In *G. arboreum*, the distribution of *GaHP* genes on chromosome A2-A06 was the highest, with five genes (*GaHP6*, *GaHP7*, *GaHP8*, *GaHP9* and *GaHP10*). There were four *GaHP* genes on chromosome A2-A05, three *GaHP* genes on chromosome A2-A10, and two *GaHP* genes on chromosomes A2-A09, A2-A11 and A2-A13; chromosomes A2-A02, A2-A07 and A2-A12 had one *GaHP* gene. In *G. raimondii*, 18 *GrHP* genes were distributed on seven chromosomes, among which two chromosomes, D5-D09 and D5-D10, were the most widely distributed; four *GrHP* genes were also present, and chromosome D5-D08 contained only one *GrHP* gene. There were three *GrHP* genes distributed on chromosome D5-D06, and two *GrHP* genes were distributed on other chromosomes, i.e., D5-D07, D5-D011 and D5-D13. In *G. hirsutum*, 30 *HP* genes were distributed on 14 chromosomes, and chromosome AD1-A06 had four *HP* genes (*GhHP3*, *GhHP4*, *GhHP5* and *GhHP6*). One *GhHP* gene was distributed on each chromosome, e.g., AD1-A12 and AD1-A30, while rest of chromosomes had two or three *GhHP* genes. 34 *GbHP* genes were distributed on 14 *G. barbadense* chromosomes. Five *GbHP* genes were distributed on chromosome AD2-D06. There were four *GbHP* genes on chromosome No. A06.

**Fig. 3:**
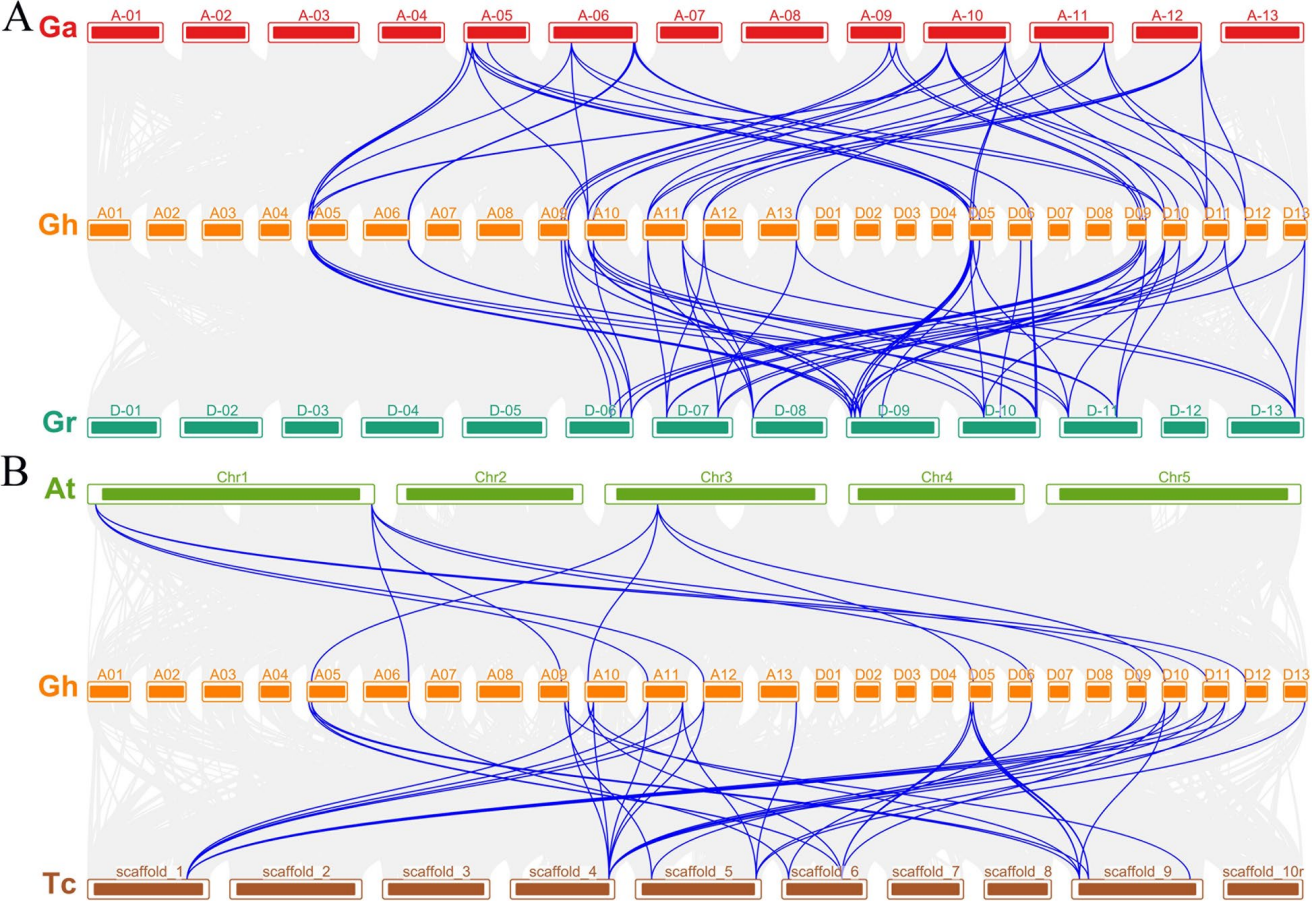
The collinear relationship *HP* genes. **A** Between *G. arboreum* and *G. hirsutum* and between *G. raimondii* and *G. hirsutum* (from top to bottom). The gray lines: the collinearity of the whole genome among cotton. The blue line: the collinearity of *HP* pairs in inter-genomics. **B** Between *Arabidopsis thaliana* (*At*), *G. hirsutum* (*Gh*) and *Theobroma cacao* (*Tc*) (from top to bottom). The gray lines: the collinearity of the whole genome among cotton. The blue line: the collinearity of *HP* pairs in inter-genomics

Gene duplication relationship analysis of *HP* gene family members from four cotton species results into 137 homologus/orthologus pairs demonstrating a good collinearity among them. Majority of duplicated pairs experienced WGD and segmental duplication with total count of 134 from 137 while only three pairs contributed from tandem duplication within the GhAt/GhDt, GhAt/A2 and A2/GhAt subgenomes. Results demonstrating the origination of ortho/paralogus from WGD before polyploidization during the time course of evolution (Fig. 3A and Table S4). We further examined the collinearity among orthologus of *G. hirsutum, T. cacao* and *A. thaliana* to explore the possible evolutionary connections among them which shows the great conservation among cotton species (Fig. 3B).

### Expression patterns of *GhHP* genes in different tissues

**Fig. 4:**
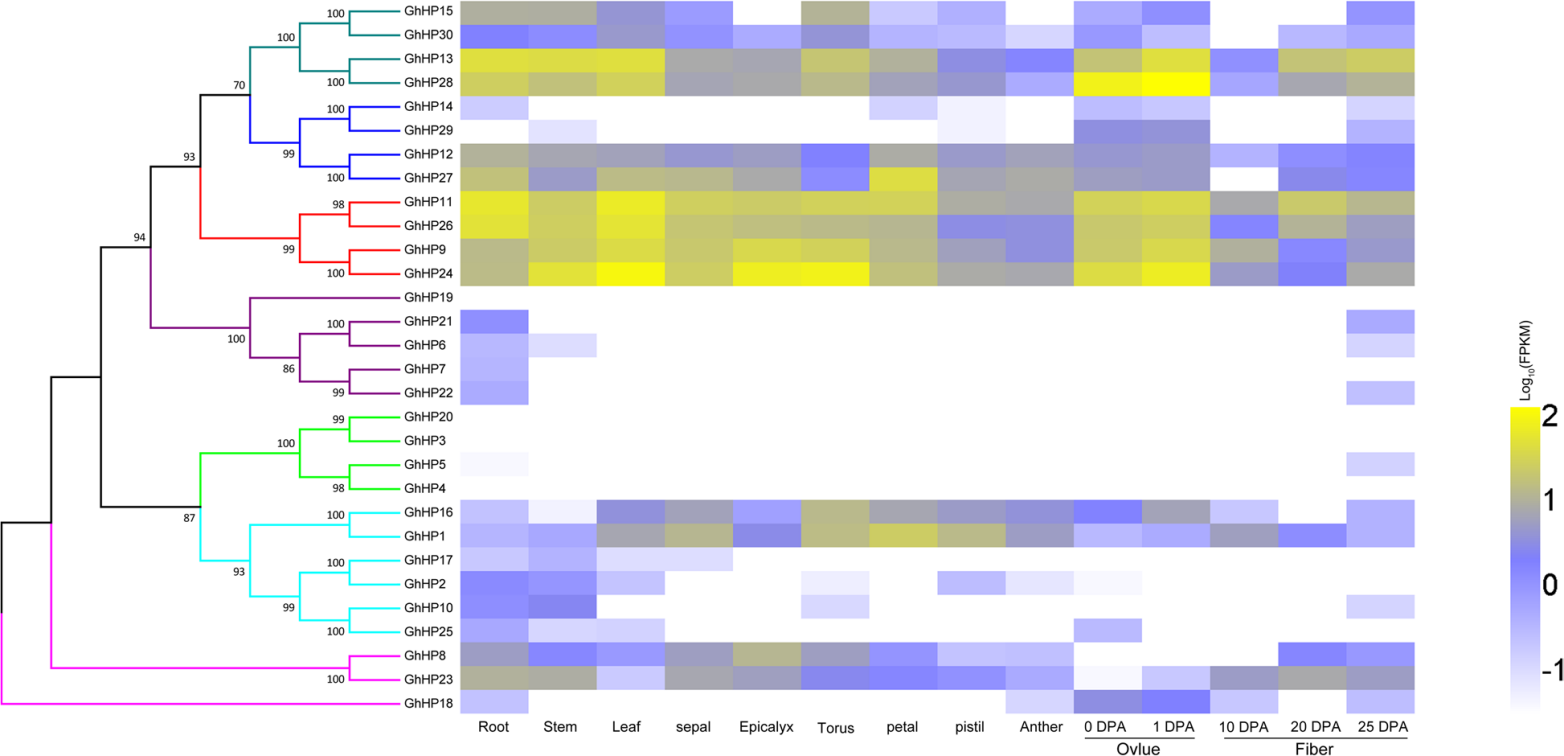
Transcriptome analysis of *GhHP* genes in different tissues

We analyzed the transcriptome data of the *HP* gene family members in various tissues of cotton to explore their biological functions and we found that *GhHPs* in subfamilies VII and IV were hardly expressed in various tissues (Fig. 4). Among them, *GhHP13*, *GhHP28*, *GhHP11*, *GhHP26*, *GhHP9* and *GhHP24* were mainly found expressed in the leaves, stems and roots, and the expression level in the ovule and fiber was also relatively high. *GhHP12* and *GhHP27* were mostly expressed in roots, leaves and petals. *GhHP15* and *GhHP23* were mainly expressed in roots, stems and the torus. This showed that these genes are not only tangled in the regulation of the vegetative growth of the above and belowground parts of cotton but are also implicated in the reproduction. Similar expression patterns were observed in same clades while significant difference can be observed among different clades especially monophyletic clades are more diverse in expression patterns.

**Fig. 5:**
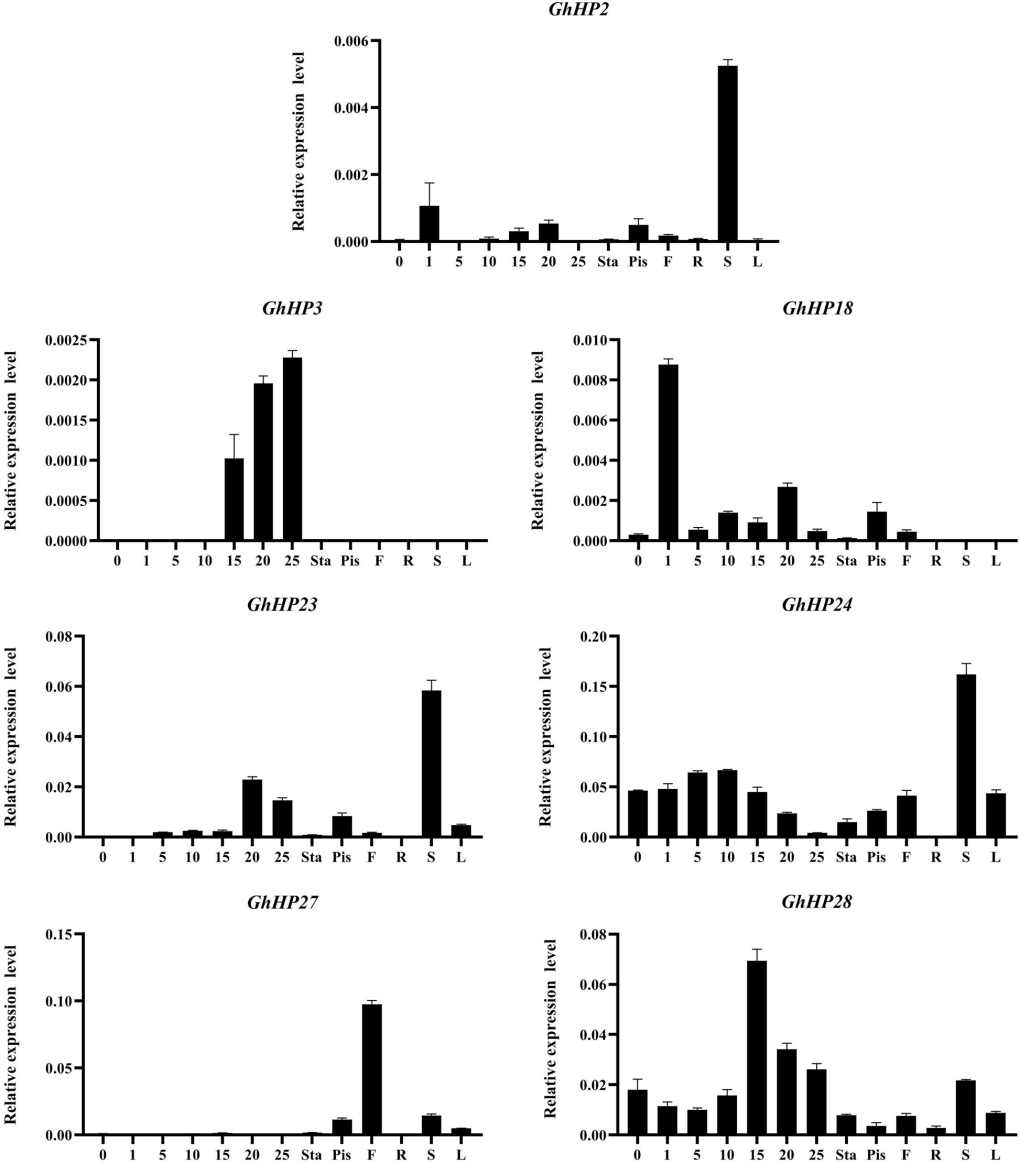
qRT-PCR was conducted to analyze the relative expression of seven *GhHP* genes in different tissues (S: stem, R: root, L: leaf, F: flower, Sta: stamen, Pis: pistil and fiber (0, 1, 5, 10, 15, 20, 25 day post anthesis)

For verification of the RNA-seq data, the qRT-PCR of seven selected *HP* genes in *G.hirsutum* was performed to analyze the expression patterns in the stem, roots, leaves, flowers, ovules (0, 1), fiber (5, 10, 20, 25DPA), stamens and pistil (Fig. 5). The results showed that three *GhHP* genes (*GhHP2*, *GhHP23* and *GhHP24*) displayed higher expression in the stem. Two *GhHP* genes (*GhHP42*, *GhHP27*) were highly expressed in the flower. Six *GhHP* genes (*GhHP2*, *GhHP3*, *GhHP18*, *GhHP23*, *GhHP24* and *GhHP28*) were constitutively expressed during fiber development. It is noteworthy that *GhHP3* was specifically expressed in fiber development but in early stages in fact its expression increases with increase in elongation of fiber development and secondary wall-thickening stage. These findings indicated the imperative roles of *GhHP* genes in fiber development. In addition to *GhHP3* gene, six more *GhHPs* were responsive in limited quantity in stamen and pistil of cotton. The results were consistent with the RNA-seq data.

### Promoter analysis of *GhHP* genes

**Fig. 6:**
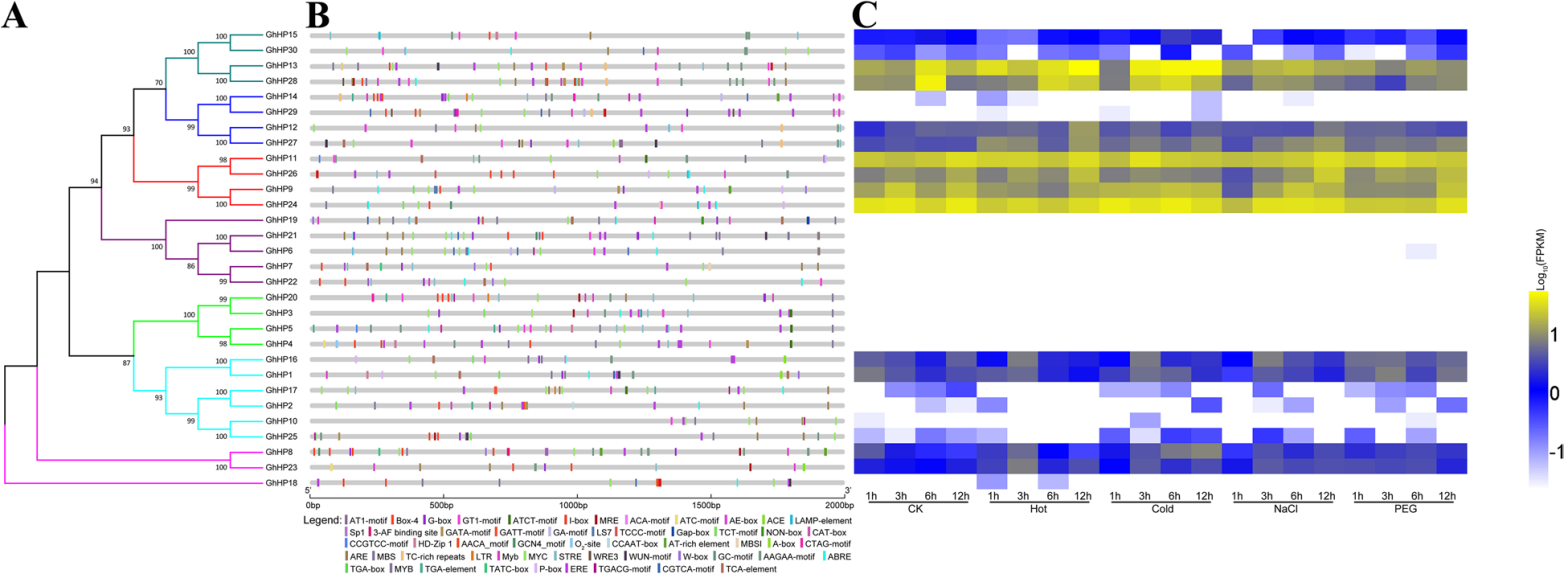
Analysis of *HP* genes promoter and its expression pattern under different stresses. **A** Phylogenetic tree of *GhHPs*. **B** *Cis*-elements in promoters of *GhHPs*. **C** Expression pattern of *GhHPs* under different stresses

Presence of *cis*-acting elements in the promoter region of genes has played an important role in the regulation of downstream genes. The 2000 bp upstream promoter sequences of 30 cotton *HP* family genes were analyzed (Table S4). These *cis* elements were mainly responsive to biotic and abiotic stress, plant hormone response, growth and development and other processes. Among the biotic and abiotic stress responses, the number of *cis*-acting elements such as Myc, ARE, STRE, MBS and LTR was the highest, indicating that the downstream genes regulated by them can respond to stress under adverse conditions to regulate gene expression to adapt to adverse environmental conditions. The presence of these *cis*-acting elements in the promoter sequences of genes such as *GhHP13*, *GhHP23*, *GhHP26*, *GhHP27* and *GhHP28* may regulate their adaptation to stressors such as temperature, salt and drought (Fig. 6).

In response to plant hormone stress, the number of the *cis*-acting element ERE in response to ethylene that of the *cis*-acting element MYB in response to auxin, and that of the *cis*-acting element ABRE and AAGAA-motif in response to abscisic acid were the highest. These *cis*-acting elements in response to hormones controls the expression of genes in response to hormone stress, thereby regulating PGD or adapting to external environmental conditions. For example, the genes (*GhHP3*, *GhHP6*, *GhHP8*, *GhHP11*, *GhHP13*, *GhHP15*, *GhHP21*, *GhHP24*, *GhHP28* and *GhHP30*) containing the *cis*-acting element ABRE and AAGAA-motif of abscisic acid regulate the PGD in response to adverse environmental conditions.

Among the *cis*-acting elements that regulate PGD, most of them are light-regulated *cis*-acting elements, with the largest number of Box-4, G-box and GT1-motifs. The *HP* family genes regulate the PGD through these light-responsive *cis*-acting elements. However, there are also some specific *cis*-acting elements involved in PGD, such as *cis*-acting elements in the *GhHP5*, *GhHP11*, *GhHP14* and *GhHP19* gene promoters involved in the specific expression of meristems (NON-box, CAT-box and CCGTCC-motif) and *cis*-acting elements in the *GhHP8*, *GhHP18*, *GhHP21 GhHP24* gene promoters that are specifically expressed in the plant endosperm (AACA-motif and GCN4-motif).

### Transcriptome based Expression profiling of *GhHPs* and validation by qRT-PCR

Considering the promising functions of *GhHP* genes under various environmental constraints, the transcriptome data of 30 *GhHP* genes under different stresses (high temperature, low temperature, NaCl treatment and PEG treatment) were analyzed [17], and different *GhHP* genes showed different responses to stress treatments (Fig. 6C). In this study, salt-tolerant materials (Zhong9807) and salt-sensitive materials (ZhongJ0102) were selected as a group to study the expression patterns of *HP* family genes under salt stress. Drought tolerant materials (ZhongH177) and drought sensitive materials (ZhongS9612) were selected as a group to study the expression patterns of *HP* family genes under drought stress. Compared with the control treatment, *GhHP23* and *GhHP27* showed up-regulated expression under various treatment conditions. This showed that these two genes have a wide range of resistance to adversity stress and can be used as candidate genes for the study of cotton resistance to stress. In addition, the expressions of *GhHP13* and *GhHP28* were up-regulated compared with the control under cold/heat treatment conditions. *GhHP26* is down-regulated under cold stress conditions. These three genes are sensitive to temperature and can be used as candidate genes for research on cold tolerance or high-temperature tolerance.

**Fig. 7:**
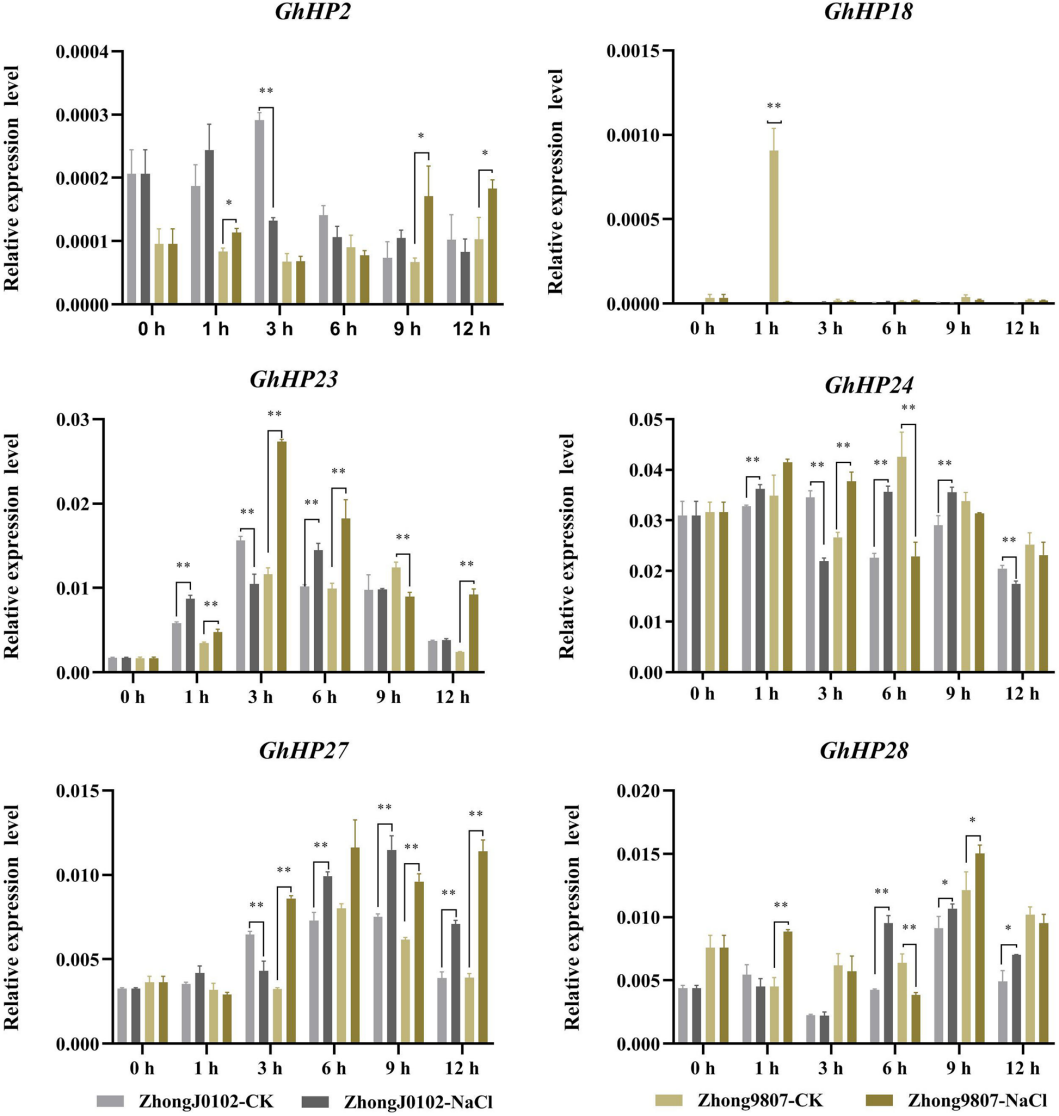
Expression levels of 6 *GhHPs* in 400 mM NaCl stress. Error bars represent SD of three independent experiments. The data represent the means _ SE from three independent experiments, t-tests: * *p* < 0.05, ** *p* < 0.01

**Fig. 8:**
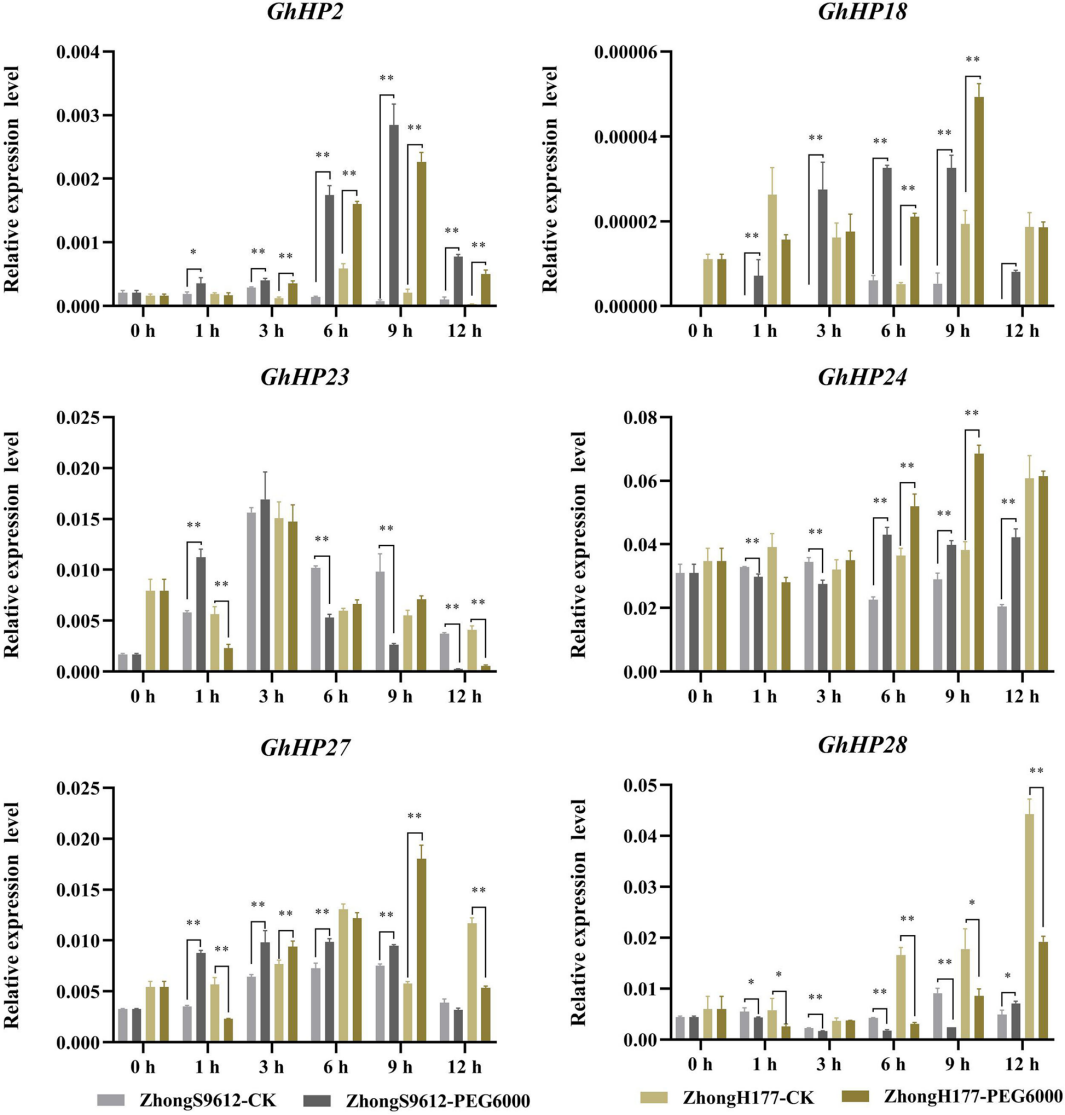
Expression levels of 6 *GhHPs* in 20% PEG6000 drought stresses. Error bars represent SD of three independent experiments. The data represent the means _ SE from three independent experiments, t-tests: * *p* < 0.05, ** *p* < 0.01

We selected a group of salt-tolerant and salt-sensitive materials (Fig. 7) and a group of drought-tolerant and drought-sensitive materials (Fig. 8) and tested whether the *HP* genes were involved in drought and salt stress tolerance of cotton using the qRT-PCR method. A total of six genes were selected for expression analysis in the two sets of materials under salt and drought treatment (Figs. 7 and 8). The results showed that the expression levels of *GhHP23*, *GhHP24* and *GhHP27* changed significantly after 3 hours of salt treatment, i.e., decreased significantly in the salt-sensitive materials and increased significantly in the salt-tolerant varieties. *GhHP24* and *GhHP28* also showed significant changes in this group of materials after 6 h of salt treatment: the expression levels of *GhHP24* and *GhHP28* increased significantly in salt-sensitive materials but decreased significantly in salt-tolerant varieties (Fig. 7). These results indicated that *GhHP* genes may be involved in regulating the salt stress response of cotton, and *GhHP23*, *GhHP24*, *GhHP27* and *GhHP28* can be used as candidate genes for salt stress regulation in cotton. After drought treatment (Fig. 8), the *GhHP* genes showed significant differences in almost every period compared with the control, and the expression trends of drought-sensitive and drought-tolerant materials were basically the same.

It is worth noting that after 1 h of PEG6000 treatment, the expression levels of the *GhHP23* and *GhHP27* genes showed significant differences between drought-sensitive and drought-tolerant materials. The expression levels of *GhHP23* and *GhHP27* decreased significantly in drought-tolerant materials and increased significantly in drought-sensitive materials (Fig. 8). These results indicate that the *GhHP23* and *GhHP27* genes are involved in the regulation of drought stress in cotton, which is consistent with the RNA-seq data.

### Interaction network of GhHP proteins

**Fig. 9:**
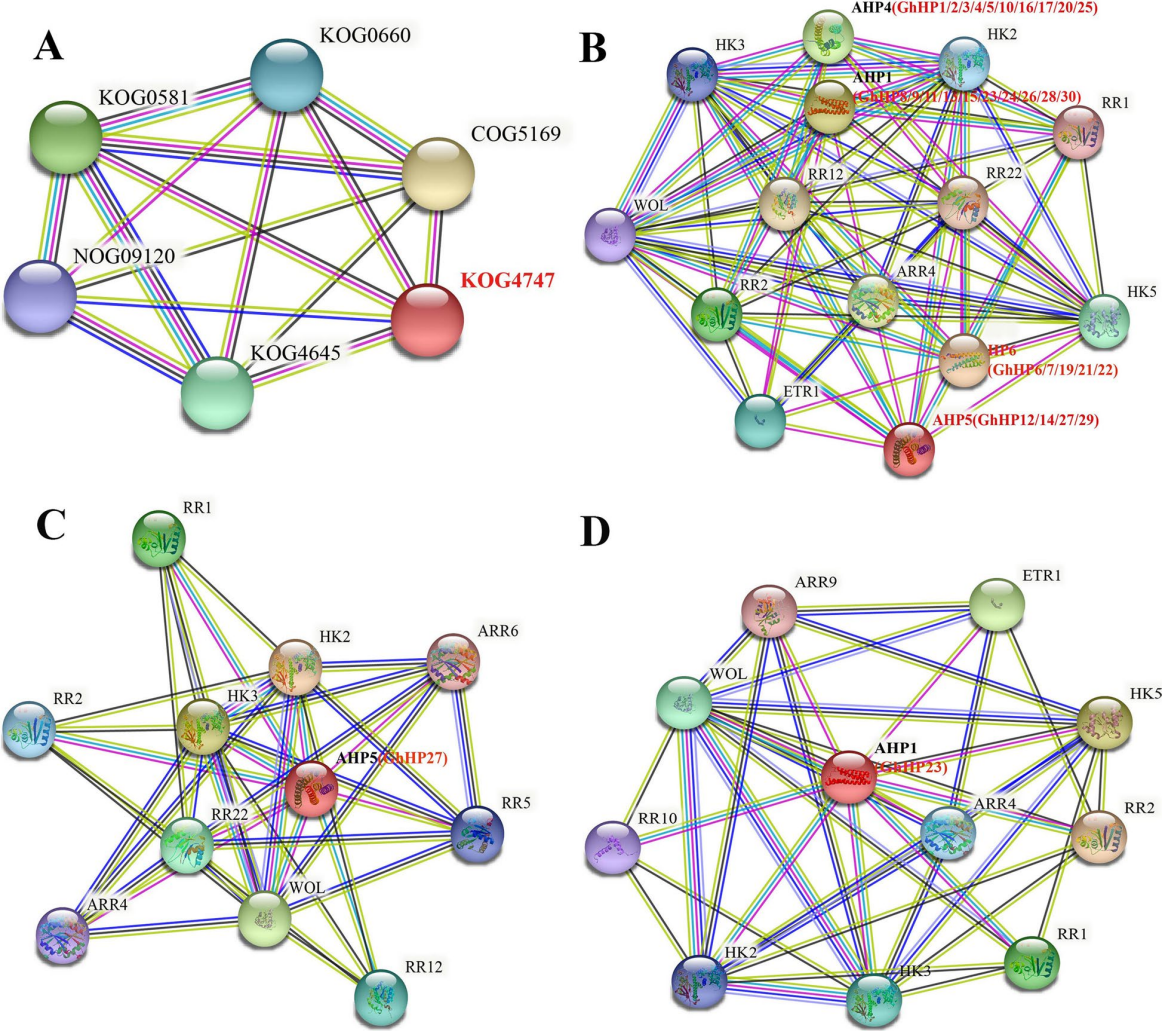
Interaction network of HP proteins. **A** Interaction network of HP proteins families in *Arabidopsis*. The red letters represent HP proteins signaling pathway. **B** Interaction network of GhHPs proteins with other proteins. The black and red letters represent AtHP proteins and cotton HP proteins, respectively. **C** Interaction network of GhRR27 proteins with other proteins. The black and red letters represent AtHP proteins and GhHP27 proteins, respectively. **D** Interaction network of GhHP23 proteins with other proteins. Black letters represent AtHP protein. Red letters represent GhHP23 protein, respectively

On the basis of homologs of Arabidopsis, we draw a protein-protein interaction network for GhHPs using online STRING database to find the key potential regulatory genes from *GhHP* gene family [13]. Based on the fully studied *Arabidopsis* HP proteins, we can infer the large part of the regulatory network involved in cotton HP proteins. Through a protein family search, we found that *Arabidopsis* HP participates in the regulation of MAP kinase cascades as a two-component phosphate layer intermediate (KOG4747) (Fig. 9A). This shows that the function of cotton HP members depends on the two-component signal transduction system and serine/threonine protein kinase signal transduction. Based on multiple sequence searches (Fig. 9B), All GhHP family proteins only interacted with HK proteins and RR proteins, which confirmed that GhHP family proteins played an important role in TCS signal transduction system. Combined with the results of qRT-PCR, we analyzed the protein interaction network of GhHP27 (Fig. 9C) and GhHP23 (Fig. 9D). In the prediction results of protein interaction network of GhHP27 and GhHP23, we found that HP27 and HP23 interacted with ARR1, ARR4, ARR5, ARR10, ARR12, AHK2, and AHK3, and these genes that transcribe these proteins are confirmed to be related to drought stress in *Arabidopsis* [18–22], indicating that GhHP27 and GhHP23 may participate in drought stress and play an important role with cotton.

### Silencing of *GhHP23* and *GhHP27* compromises cotton tolerance to drought and salt stress

**Fig. 10:**
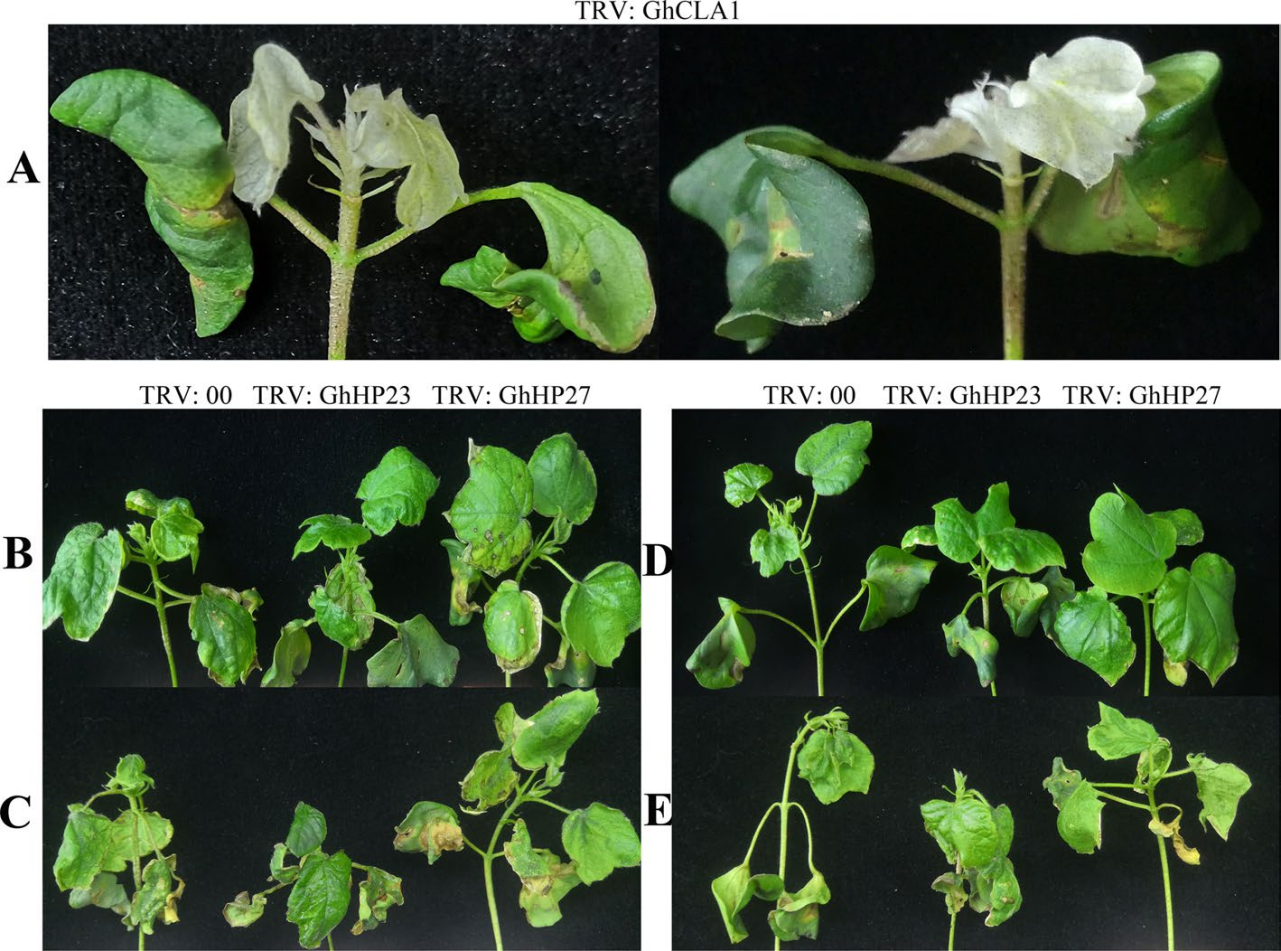
Silencing of *GhHP23* and *GhHP27* compromised cotton drought stress tolerance. **A** The positive control plants. **B** and **C** Phenotypes of TRV: 00, TRV: GhHP23 and TRV: GhHP27 before and after 400 mM NaCl treatment. **D** and **E** Phenotypes of TRV: 00, TRV: GhHP23 and TRV: GhHP27 before and after 20% PEG6000 treatment

**Fig. 11:**
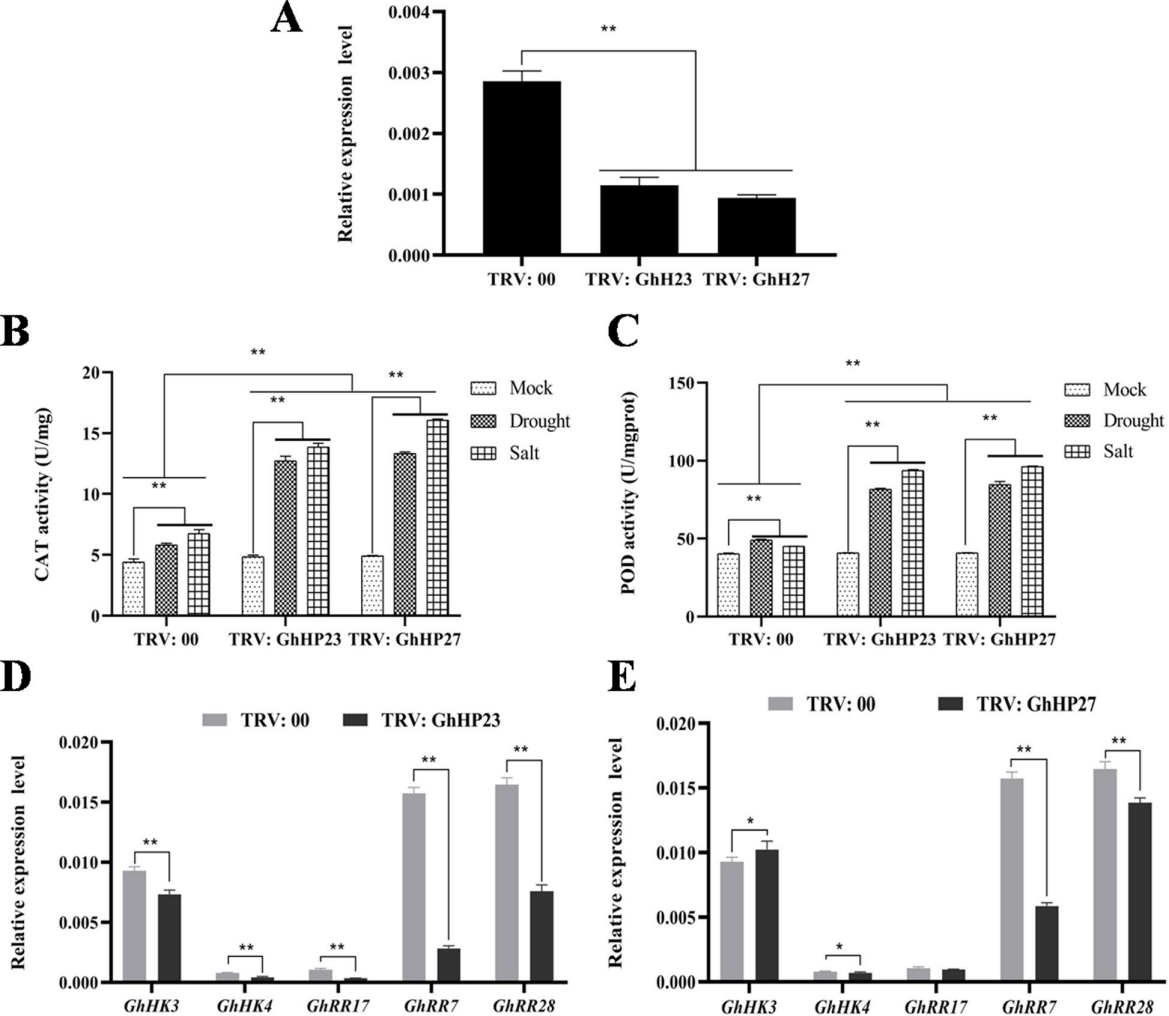
Indicator determination after Silencing of *GhHP23* and *GhHP27* under drought stress tolerance. **A** Relative expression levels of control plants and target gene-silenced. **B** CAT activity between control plants and target gene-silenced under TRV: 00, 400 mM NaCl and 20% PEG6000 stress treatment. **C** POD activity between control plants and target gene-silenced under TRV: 00, 400 mM NaCl and 20% PEG6000 stress treatment. **D** Expression levels of *GhHK3*, *GhHK4*, *GhRR17*, *GhRR7* and *GhRR28* genes in TRV: GhHP23 silenced plants. **E** Expression levels of *GhHK3*, *GhHK4*, *GhRR17*, *GhRR7* and *GhRR28* genes in TRV: GhHP27 silenced plants. The data represent the means _ SE from three independent experiments, t-tests: * *p* < 0.05, ** *p* < 0.01

Considering expression patterns of the *GhHP* genes based on transcriptome data, we organize a VIGS system to check their expression responses after knock down of two important *GhHP* genes (*GhHP23* and *GhHP27)*. TRV: GhCLA1 was used as a positive control, and its cotton leaves showed an albino phenotype two weeks after VIGS operation (Fig. 10). We did not find any significant difference in expression levels of TRV: GhHP23 and TRV: GhHP27 as compared to control one (TRV: 00). However, qRT-PCR analysis showed the declined expression levels of *GhHP23* and *GhHP27* (Fig. 11A), indicating that these two genes were silenced. Therefore, we treated the empty and silent plants with 400 mM NaCl and 20% PEG6000 and found that the silent plants exhibited a phenotype of wilting and water loss. Due to the decreased expression levels of *GhHP23* and *GhHP27*, its ability to tolerate salt stress and drought stress decreased. The results showed that *GhHP23* and *GhHP27* are positive regulators of salt and drought tolerance.

Under salt and drought stresses, the POD and CAT activities in TRV: GhHP23, TRV: GhHP27 and TRV: 00 control cotton leaves were measured (Fig. 11B/C). Compared with the TRV: 00 control cotton, TRV: GhHP23, TRV: GhHP27 cotton showed higher POD and CAT levels. These results indicate that silencing of the *GhHP23* and *GhHP27* genes enhanced the drought tolerance of the cotton plants. Based on the prediction results of the protein regulatory network involved in GhHP23 and GhHP27, we detected the expression of *GhHK3*, *GhHK4*, *GhRR17*, *GhRR7* and *GhRR28* in TRV: GhHP23, TRV: GhHP27 and TRV: 00. Compared with the control, the expressions of *GhHK3*, *GhHK4*, *GhRR17*, *GhRR7* and *GhRR28* decreased significantly in TRV: GhHP23 (Fig. 11D). However, in TRV: GhHP27 plants, the expression of GhHK3 increased and the expression of GhHK4 decreased compared with the control. The expression of GhRR7 and GhRR28 decreased significantly with the control (Fig. 11E). This shows that the silencing of *GhHPs* directly affected the expression levels of *GhHKs* and *GhRR*s.

## Discussions

As a component of TCS, *HP* genes have implications in regulating several abiotic stresses in no of crop plants. The *HP* genes have been reported in several dicot crops including Chinese cabbage [23], *Arabidopsis* [24] and soybean [25] as well as in monocot crops like maize [26]. However, the *GhHP* family genes have not been identified in cotton, and the role of *HPs* in growth, development and abiotic stress of cotton is still unclear. Present study comprises of complete systematic characterization and comparative analysis of HP gene family in multiple plant species for their different aspects from structure to function and evolution. Two *GhHP* genes are also well explored by their knockdown for their predicted roles in drought stress through VIGS analysis. This study will lay down a foundation for exploring the multiple functions of *HP* genes in upland cotton in future, especially in abiotic stress tolerance mechanism and fiber development.

Overall 30, 34, 21 and 18 *HP* family members were recognized in *G. hirsutum*, *G. barbadense*, *G. arboreum* and *G. raimondii*, respectively. Unexpectedly, no of *HP* genes in tetraploid species are not twice to their diploid progenitors but less than the expected amount demonstrating the gene loss during evolution under strong purifying selection indicating the functional conservation of *HP* gene family. Uneven distribution of the *HP* genes was observed on chromosomes of the cotton A and D subgenomes. Further, complete systematic investigations are needed to provide insight into the *HP* gene family evolution in cotton. The CDS sequences of the cotton *HP* genes ranged in size from 186 to 678. Most HP proteins are localized in the nucleus, and only a few are localized in the cytoplasm and extracellular compartment. Cotton HP proteins contain only the Hpt domain. These characteristics are consistent with the *HP* genes in Chinese cabbage [23], which indicates that the nucleic acid and amino acid characteristics are relatively conserved in the differentiation process of *HP* genes family in different species.

Gene duplication is one of the important processes for structural and functional evolution of different organisms. Whole-genome doubling (WGD) can generate huge amount of repetitive genes at the same time, which is one of the most important evolutionary events that widely exist in plants, animals and fungi [27–32]. Phylogenetic tree analysis divided the *GhHP* gene family into 7 groups/clades as shown in Fig. 1. The collinearity results showed that the expansion of the *HP* family occurred as a result of WGD/segmental duplications and tandem duplication (Table S4). In the process of evolution, gene synteny will decrease due to various factors, and the gene synteny between species with greater evolutionary distance is lower [29]. The synteny analysis of *Arabidopsis thaliana*, *T.coco* and *G. hirsutum* also proved this point, that is, *GhHPs* had good collinearity with *TcHP* but poor collinearity with *AtHP*. The phylogenetic tree analysis among different species shows that monocotyledonous plants occurred only on three branches, V, VI and VII, and dicotyledonous plants were distributed among the I-VII subfamilies. It is speculated to be associated with the ancient genome of the γ triploidization event shared with dicotyledonous plants [33, 34]. Dicotyledonous plants have undergone different lineage-specific genome-wide doubling processes [35], resulting in different evolutionary events of the *HP* gene in each species. Study of HP gene family in Arabidopsis demonstrates their implication in growth & development of organs [36–38]as well as in no of various kinds of abiotic stresses like excessive concentrations of salts, abscisic acid, water deficiency, and stress signaling [19, 20, 39, 40]. Phylogenetic tree analysis showed that *GhHP2*, *GhHP7*, *GhHP9*, *GhHP12*, *GhHP17*, *GhHP19*, *GhHP24* and *GhHP27* had the highest similarity with *AHPs* and might play similar functions in cotton.

We further analyze the different *cis*-elements present in promotor region of *GhHP* genes associated with different environmental constraints. To gain further insights into the regulation of *GhHP* genes under changing environmental conditions, we investigated the *cis*-regulatory elements inside their promoter regions. These *cis*-acting elements are mainly associated with different responses from biotic and abiotic stresses, plant hormonal responses, PGD and other processes. Our finding for *cis* elements are consistent with previously reported results [4–7]. Transcriptome data analysis of the *GhHP* family gene tissue expression and related stress expression showed the similar expression patterns exhibited by homolog genes signifying their functional conservations. The qPCR results showed that under salt stress and drought stress, *GhHP* family genes are involved in regulating abiotic stress in cotton; in particular, *GhHP23* and *GhHP27* plays a vital role in controlling salt and drought stress in cotton by analyzing the expression of *GhHP* family genes in two groups of control materials. This result is inseparable from their genetic structure. Tissues specific expression profiling illustrates the identification of specific genes and their particular role in certain developmental stage [41]. In *Arabidopsis*, several AHPs perform positive roles in regulation of cytokinin signaling [12].

The role of *HP* family genes in cotton was estimated, and preliminary verification was carried out through transcriptome data analysis and real-time fluorescence quantitative experiments of the *GhHP* family genes. The results showed that the *GhHP3* gene was expressed only in the middle and late stages of fiber development, *GhHP27* was mainly upregulated in flowers. *GhHP2*, *GhHP23* and *GhHP24* were basically upregulated in stems. *GhHP18* in the initial stage of development, and *GhHP24* and *GhHP28* were constitutively expressed mainly in each phase of fiber development. These results are consistent with the transcriptome data. This indicates that the *GhHP* family genes not only participate in regulating the vegetative growth of cotton but might be involved in playing crucial role at initial stage, elongation stage, secondary wall-thickening stage and dehydration maturity step of fiber development in cotton.

MAPK signaling pathway is well known biological process for regulating almost all aspects of PGD and manifestation of multiple responses exhibited by plants in response to environmental stimuli [42, 43]. For example, knockdown of the *GhMEKK12* and *GhRAF4* genes in cotton plants decline the drought stress resistance significantly [44]. Similarly, *GhMPK17* was reported in multiple functions in same study like ABA signaling, response to osmotic stress and high salinity stress resistance [45]. Protein regulatory network analysis showed that the HP protein is involved in the MAPK cascade regulation (KOG4747) regulatory pathway, and might have implications in MAPKKK, SSK2 and serine/threonine related protein kinases (KOG4645), mitogen-activated protein kinase (MAP2K) (KOG0581) and other regulatory pathways. This indicates that GhHPs may interact with MAP kinase and ultimately participate in the regulation of abiotic stress in cotton. Similar results are published from previous studies like which shows that interaction of *OsMAPKKK63* with *OsMKK6* and *OsMKK1* in response to salt stress and regulation of seed dormancy [46]. Similar finding were reported by [47], in which *OsMPKK10.2* regulates the activation of various MAPKs for enhancing drought and disease resistance. WRKY33/MKS1, MPK4/6 and MEKK1 participate in the regulation of salt stress response [48, 49] and *GhMPK16* shows its responses toward regulating drought and disease resistance mechanism [50].

To further explore the functions of *GhHPs* in cotton salt stress responses and drought tolerance, two *GhHPs* (*GhHP23* and *GhHP27*) were knocked down with VIGS system using TRV virus successfully. The results showed that knockdown of *GhHP23* and *GhHP27* genes reduced the salt and drought resistance in cotton plants making them more vulnerable to given stresses as treatments. Stressful environment created by drought and salinity causes the onset of production of reactive oxygen species in excess amount which will be mitigate by alternative responsive systems like superoxide dismutase or catalases [51]. We measured the physiological indexes of POD and CAT activities under salt and drought stresses. Plants silenced with activity of *GhHP23* and *GhHP27* under drought and salt stress genes were observed having increased POD and CAT activities as compared to control ones. This indicated that the scavenging ability of ROS was enhanced, which may be one of the pathways through which *GhHP23* and *GhHP27* regulate salt and drought resistance in cotton. Based on the protein regulatory network analysis of *GhHP23* and *GhHP27*, we speculated that *GhHP23* interacts with *GhHK3*, *GhHK4, GhRR7, GhRR17* and *GhRR28* and detected the expression levels of the five protein-related genes in the TRV: *GhHP23* and TRV: *GhHP27* target gene-silenced plants (Fig. 8G/H). The results showed the similar expression of *GhRR23* gene with those five genes. After *GhRR23* was silenced, the expression levels of the *GhHK3*, *GhHK4*, *GhRR7*, *GhRR17* and *GhRR28* genes were significantly reduced. After *GhRR27* was silenced, in addition to the significant increase in *GhHK3*, the *GhHK4* was significantly decreased, and the expression levels of *GhRR7* and *GhRR28* were significantly decreased. Studies have shown that in the TCS system, the HP-conserved His residue accepts a phosphoryl group from HK and moves from the cytoplasm to the nucleus, where it transports the phosphoryl to the RR containing the Asp residue [24, 52–54]. It is speculated that GhHKs interact with GhHPs and GhRRs to negatively regulate the stress of cotton salt and drought resistance. This result is consistent with the finding that *AHP2/AHP3/AHP5*, *OsAHP1* and *OsAHP2* [55] negatively regulate plant abiotic stress.

## Conclusions

It's here, a comprehensive analysis of the *HP* gene family, an important component of the TCS system involved in cytokinin signal transduction, was performed in four species of *Gossypium*. Systematic and comparative structural, functional and evolutionary analysis of *HP* gene family members provides indepth insights into their complete background and their possible use future research for provision of genetic material for plant breeding and genome editing projects. The expression profiling of *GhHPs* in diverse no of tissues under multiple abiotic stress treatments were examined, using transcriptome data signifies their possible predicted roles reproductive growth of cotton no of abiotic stress, particularly salt and drought stress. Moreover, protein regulatory network analysis and validation of two important regulatory *HP* genes (*GhHP23* and *GhHP27*) through a VIGS system supports their implications in salt and drought stress tolerance mechanisms. It may regulate the content of ROS by interacting with some genes in the *GhHK* and *GhRR* families, thereby responding to cotton salt and drought stresses.

## Materials and methods

### Identification and analysis of *HP* genes in cotton

*Arabidopsis* HP protein sequences were downloaded from TAIR (http://www.arabidopsis.org/). *Gossypium* (*G. arboreum* [56], *G. raimondii* [57], *G. barbadense* [58], and *G. hirsutum* [17]) HP protein sequences were downloaded from CottonFGD (https://cottonfgd.org/about/download.html) [59]. Other plant species including *Zea mays*, *Populus trichocarpa*, *Oryza sativa*, *Glycine max*, *Theobroma cacao* and *Vitis vinifera* HP protein sequences were downloaded from phytozome (https://phytozome.jgi.doe.gov/pz/portal.html).

Identification of genes encoding proteins containing HP-domains was performed using Interproscan 5 (http://www.ebi.ac.uk/interpro/) [44, 60] against the HP domain (IPR008207) (e-value<0.1). Blastp search for HP domain and protein sequence of several *HP* genes from *Arabidopsis* were used as query to extract all the possible *HP* genes in cotton. Web server of SMART (http://smart.embl-heidelberg.de/) was used to recognize the HP domain [44]. We get the physicochemical properties of *HP* gene family cotton members from ExPASy (http://web.expasy.org/) following subcellular localization prediction by the Cello v2.5 server [61].

### Sequences alignments and phylogenetic analysis

DNA Man 2.0 was used for multiple sequence alignment HP domain sequences of four cotton species. HP proteins were aligned for sequence logo analysis, and WEBLOG was used for submission of multiple alignment result to generate the logos [62]. We use MEGA7 for multiple sequence alignment of HP proteins sequences from all eleven plant species using neighbor-joining method with 1000 bootstrap value under Poisson model [63] representing the evolutionary connections of all classes of plants.

### Analysis of the gene structure, conserved motifs and chromosomal locations

Multiple Em for Motif Elicitation website (http://meme-suite.org/) was employed to recognize the conserved motifs of *HP *genes with p-value less than 1e^−5^ [64]. Gene Structure Display Server [65] for gene structure analysis. We used TBtools for graphical representation of conserved motifs, gene structure and chromosomal locations for each gene [66]. Gene duplications analysis was calculated with advanced Circos and multiple collinearity of duplicated gene was displayed with TBtools software to show their relationship with each other [66].

### Promoter analysis and Expression profiling of *HP* Genes

2000 bp upstream nucleotide sequences of *HP* gene family members were acquired from the CottonFGD as promoters [67] and submitted to PlantCARE database (http://bioinformatics.psb.ugent.be/webtools/plantcare/html/) to find the *Cis* elements of related functions. RNA-seq data was accessed through Cotton Omics Database (http://cotton.zju.edu.cn/10) for expression profiling of GhHPs under different circumstances [17] following the construction of heat map and *Cis*-elements representation along the tree used TBtools.

### qRT-PCR of GhHPs

Plant material for upland cotton verities “Zhong9807, ZhongJ0102, TM-1, ZhongH177, and ZhongS9612” were obtained from the parent lab (Institute of Cotton Research, CAAS, China). For qRT-PCR, based expression analysis seeds of *G. hirsutum cv* Zhong9807 (Salt tolerant), ZhongJ0102 (Salt sensitive), ZhongH177 (Drought tolerant) and ZhongS9612 (Drought sensitive) were grown in controlled condition (25 °C, 16 h/8 h day/night) and treated with 200 mM NaCl and 12% PEG along with zero treated plants at age of 4 weeks older with exception of only TM-1 seeds which were grown at 28 °C and ultimately samples were collected from roots, stem and leaves for extraction of total RNA. Similarly, samples were also taken after 1, 3, 6, 9, 12, 15, 18, 21, 27 days of flowering, fiber and stamen pistil and saved in liquid nitrogen following transfer into -80°C for future use and was reverse transcribed using PrimeScript™ kit. Using the Bio-Rad CFX96 fluorescence quantitative PCR platform with TB Green® Fast qPCR Mix, we performed the qRT-PCR analysis with 3 independent replications, and 2^−ΔΔCT^ [68] system was used to measure the relative expressions of *GhHP* genes and *GhUBQ7* (GenBank accession No.DQ116441) was used for internal references. Specific primers used for qRT-PCR of *GhHPs* were given in Table S1.

### PPI Network of *HP* Gene family on homologs basis

Using 0.4 threshold as a confidence level, we used the online database of the STRING (htps://string-db.org/) to investigate the interaction of HP proteins on the basis of *Arabidopsis* orthologues.

### Vector construction of cotton VIGS system

322bp and 215bp sequences of *GhHP23* and *GhHP27* genes were amplified using cDNA from Zhong9807 of upland cotton and inserted into TRV plasmid vector by using a single step cloning kit (Vazyme biotech, China) to generate vector gene complexes for both genes TRV: GhHP23 and TRV: GhHP27. The empty vector TRV: 00 as a control. GV3101 strain of *A. tumefaciens* was used for transformation of constructs by electroporation method. For whole process of VIGS, we followed Wang et al. [60]. Gene specific primers were used for VIGS as given in Table S1.

### Salt and drought tolerance for functional analysis of *GhHP* genes

Seedlings from Zhong9807 were grown in growth chamber under favorable conditions (25 °C, 16 h/8 h light/dark period). The efficacy of VIGS was verified by qRT-PCR analysis. The roots of both kind of plants from control group and plants with knockdown gene were watered with 400 mM NaCl up to 24 h with three replications and each replication consists of 15 plants. 20% PEG6000 as drought stress treatment uses the same methods and steps as described above.

### Determination of salt and drought stress-related physiological parameters

The peroxidase (POD) activities of TRV: 00, TRV: GhHP27 and TRV: GhHP23 was measured by using POD kit (Solarbio, BC0170, Beijing, China) according to directions given in kit manual and catalase (CAT) activity was computed refer to the description of Zhang et al., [44].

## Supplementary Information

**Additional file 1: Table** **S1.** The primers used for qRT-PCR in this study. **Table** **S2.** Information of *HP* genes in this study. **Table** **S3.** The biophysical properties of cotton *HP* genes in this study. **Table** **S4.** Collinearity analyses of *HP* gene family in cotton species. **Table** **S5.** Analysis of GhHPs *cis*-acting elements. **Table** **S6.** RNA-seq data analysis of *GhHPs *expression profiling in different tissues. **Table** **S7.** RNA-seq data analysis of *GhHPs* expression profiling in different stresses.

**Additional file 2: Fig.** **S1.** Comparison of the gene structure and domains in *HP* genes on the *G*. *hirsutum* and *Arabidopsis*. **Fig.** **S2.** Comparison of the gene structure and domains in *HP* genes on the *G. barbadense* and *Arabidopsis*. **Fig.** **S3.** Comparison of the gene structure and domains in HP genes on the *G. arboreum* and *Arabidopsis*. **Fig.** **S4.** Comparison of the gene structure and domains in *HP* genes on the *G. raimondii* and *Arabidopsis*. **Fig.** **S5.** Chromosomal localization of 103 HP genes in four cotton species.

## Data Availability

The genomic data of cotton, Arabidopsis thaliana in the article can be downloaded from Cotton FGD (https://cottonfgd.org/) and TAIR (https://www.arabidopsis.org/) respectively. Other plant species includes *Zea mays*, *Populus trichocarpa*, *Oryza sativa*, *Glycine max*, *Theobroma cacao* and *Vitis vinifera* HP protein sequences were downloaded from phytozome (https://phytozome.jgi.doe.gov/pz/portal.html). The analysis software, analysis methods and datasets generated are available from the corresponding author on reasonable request.

## Abbreviations

HP: histidine phosphotransfer
PGD: plant growth development
TCS: Two-component system
HK: histidine kinase
RR: response regulators
*Os*: *Oryza sativa*
*At*: * Arabidopsis thaliana *
*Gh*: *G. hirsutum*
*Ga*: *G. arboreum*
*Gb*: *G. barbadense*
*Gr*: *G. raimondii*
*Tc*: *Theobroma cacao*
*Pt*: *Populus trichocarpa*
*Vv*: *Vitis vinifera*
*Zm*: *Zea mays*
*Gm*: *Glycine max*
HMM: Hidden Markov model
pIs: isoelectric points
MWs: Molecular weights
NJ: Neighbor joining
VIGS: Virus-induced Gene Silencing
POD: Peroxidase
CAT: Catalase
WGD: Electrospray ionization
qRT-PCR: Quantitative real-time polymerase chain reaction
