# Supplementary material for: Systematic analysis of Histidine photosphoto transfer gene family in cotton and functional characterization in response to salt and around tolerance
Source: BMC Plant Biol. 2022 Nov 28;22:548. doi: 10.1186/s12870-022-03947-5 (PMC9703675; doi:10.1186/s12870-022-03947-5)

Fig. S1 Comparison of the gene structure and domains in *HP* genes on the *G. hirsutum* and *Arabidopsis*.


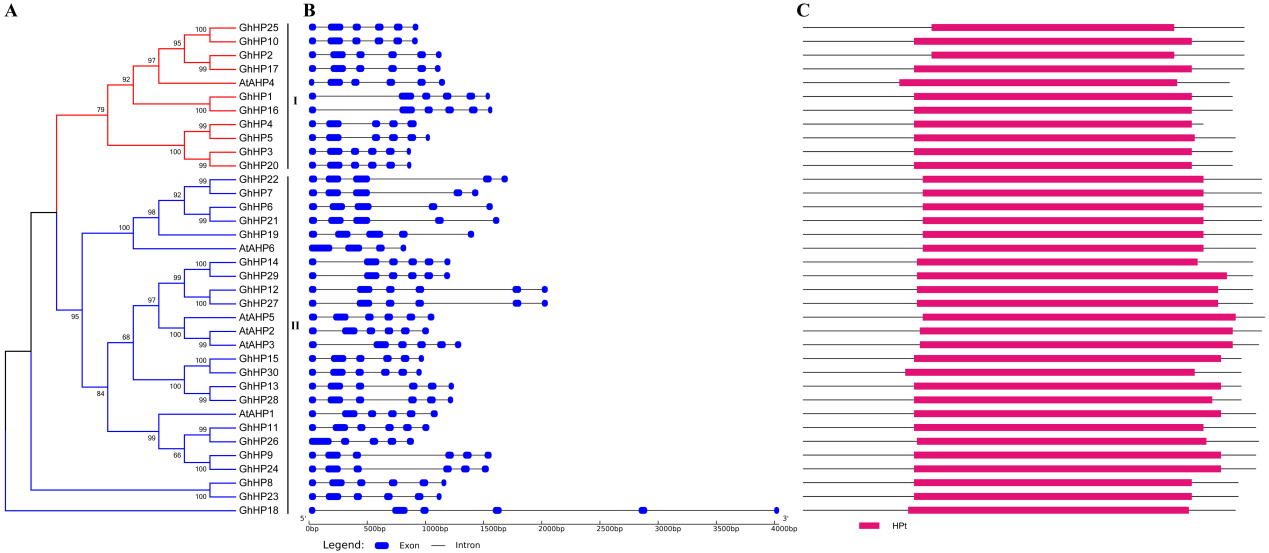


Fig. S2 Comparison of the gene structure and domains in *HP* genes on the *G. barbadense* and *Arabidopsis*.


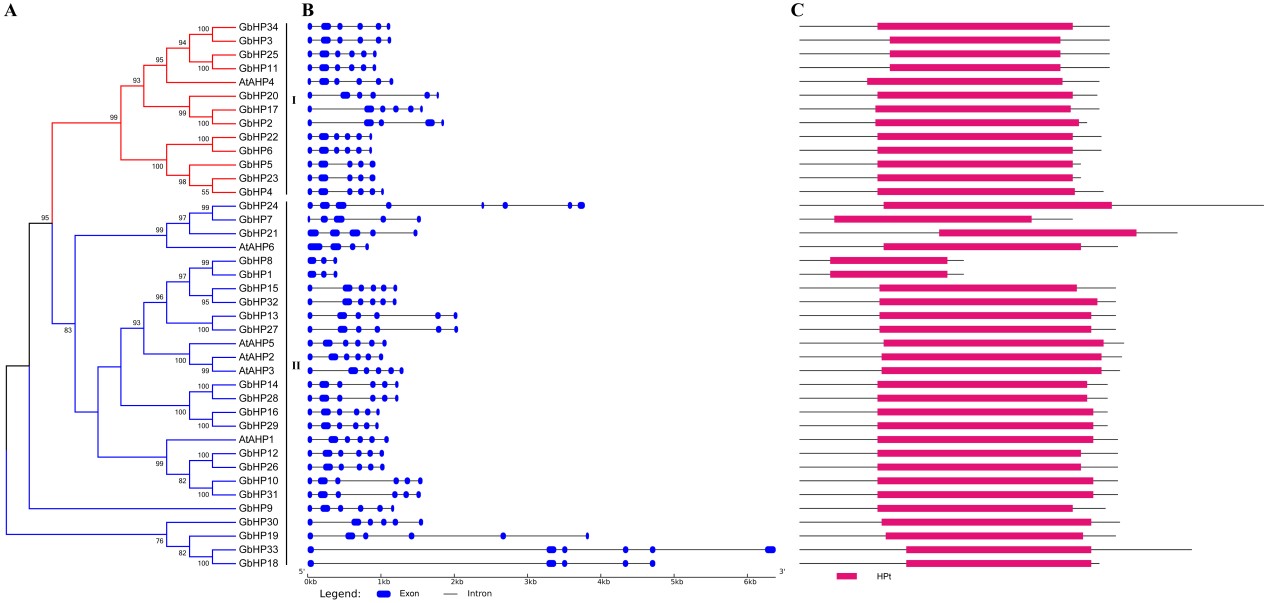


Fig. S3 Comparison of the gene structure and domains in *HP* genes on the *G. arboreum* and *Arabidopsis*.


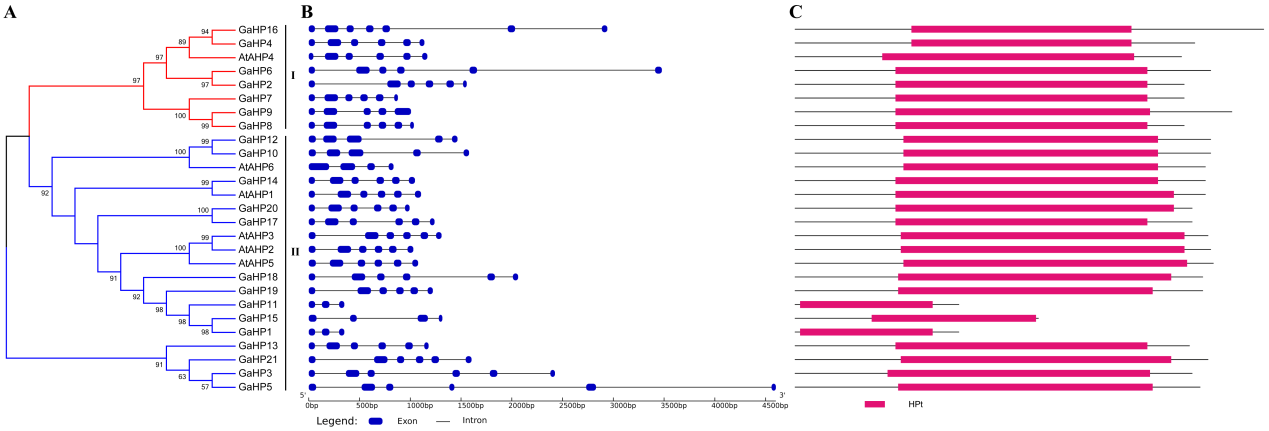


Fig. S4 Comparison of the gene structure and domains in *HP* genes on the *G. raimondii* and *Arabidopsis*.


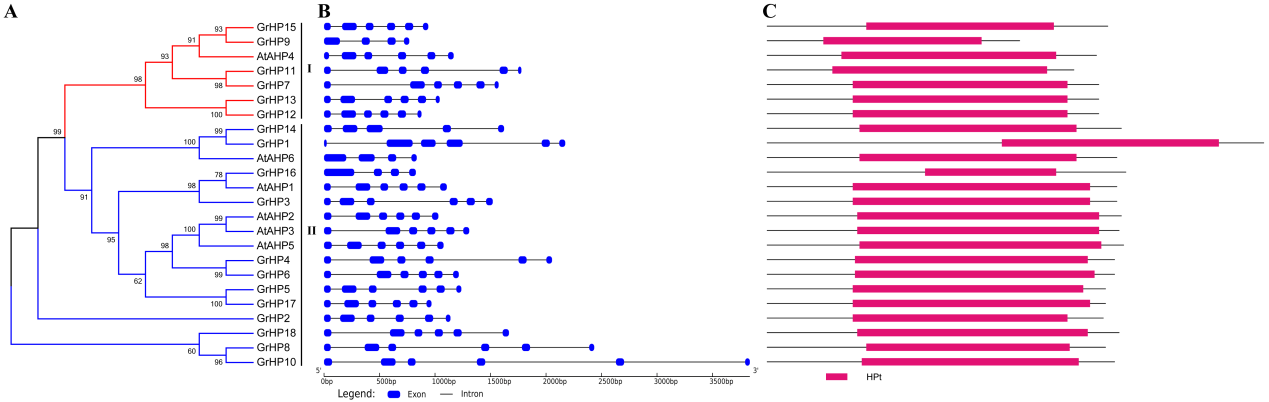


Fig. S5 Chromosomal localization of 103 *HP* genes in four cotton species.


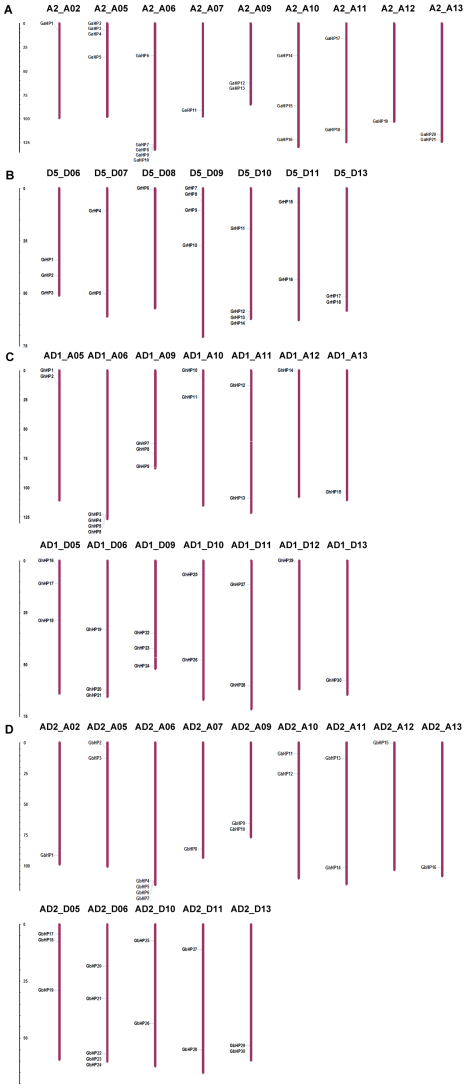

Supplement: Supplementary file 2 — Additional file 2: Fig. S1. Comparison of the gene structure and domains in HP genes on the G. hirsutum and Arabidopsis. Fig. S2. Comparison of the gene structure and domains in HP genes on the G. barbadense and Arabidopsis. Fig. S3. Comparison of the gene structure and domains in HP genes on the G. arboreum and Arabidopsis. Fig. S4. Comparison of the gene structure and domains in HP genes on the G. raimondii and Arabidopsis. Fig. S5. Chromosomal localization of 103 HP genes in four cotton species. [file 12870_2022_3947_MOESM2_ESM.docx]
